# Assessment of Impact of the ARIADNE Research: Insights Into Improving Access in Mental Health

**DOI:** 10.1192/bjo.2025.10082

**Published:** 2025-06-20

**Authors:** Rahul Bhattacharya, Emanuele Valenti, Domenico Giacco

**Affiliations:** ^1^East London NHS Foundation Trust, London, United Kingdom; ^2^Warwick Medical School, Warwick, United Kingdom

## Abstract

**Aims:** Enduring inequalities in mental healthcare exist between UK minority ethnic and White British groups, which were further aggravated during the pandemic. Through 2022–23 the nationally funded ARIADNE research project carried out qualitative research and co-production workshops to suggest local (in four participating sites of England) and also identified over-arching solutions to improve access and experience of care. After the ARIADNE research project ended, a further co-designed *impact analysis* initiative was carried out in 2024 in two original participating sites (Coventry/Warwickshire and East London).

**Methods:** Workshops were held in the two sites, attended by staff and experts by experience (carers and service users) to explore the impact and progress of the *action plans* from the ARIADNE study. Subsequently a national workshop was then held bringing together national opinion leaders and local stakeholders to identify key themes.

**Results:** A content analysis of the workshops and the national event minutes were carried out to identify progress, ongoing barriers and solutions to improving access:

There is a need to refine the concept of minoritised communities. Sharing experiences of racism towards individuals from *minority ethnic groups who grew up in England* and *towards immigrants* would be valuable. Care providers should arrange safe spaces for these conversations.

Pandemic and lockdown deteriorated the quality of mental health care provision and increased demand for mental health support. This disproportionately affected ethnic minorities and exacerbated their struggle in accessing mental healthcare complicated by *stigma* (both internal, in-group, external and cultural).

Professionals were in some cases experienced as being ‘blind’ to the issues of ethnic minorities and also impacted by institutional racism.

Education, cultural mediation and digital interventions that can offer solutions and overcome barriers to access the solutions need to be local and personalised.

Crucially, a human rights approach is required to promote integration and social cohesion. Offer of care should be diversified by including participatory culture, voluntary sector involvement and lived-experience involvement (e.g. peer work). Some potentially helpful developments and service reconfigurations were noted with population-based approach and neighbourhood models of community mental health care.

**Conclusion:** Locally led co-production research offer valuable intelligence and can be a resource to local health systems. It can utilised in planning of service re-design and resource allocation. Such continuous co-production increases research impact and minimises delay in putting research findings into practice. The themes raised and initiatives undertaken may be inspirational to other areas and national initiatives.

